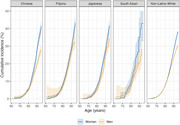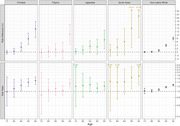# Sex/gender differences in lifetime dementia risk among Asian American ethnic groups and non‐Latino White older adults in California

**DOI:** 10.1002/alz70860_107002

**Published:** 2025-12-23

**Authors:** Liliana Paloma Rojas‐Saunero, Yingyan Wu, Yixuan Zhou, Eleanor Hayes‐Larson, Holly C Elser, Alexander Ivan B. Posis, Alka M. Kanaya, Rachel A. Whitmer, Paola Gilsanz, Elizabeth Rose Mayeda

**Affiliations:** ^1^ UCLA Fielding School of Public Health, University of California, Los Angeles, CA, USA; ^2^ Leonard Davis School of Gerontology, University of Southern California, Los Angeles, CA, USA; ^3^ Hospital of the University of Pennsylvania, Philadelphia, PA, USA; ^4^ University of California, Davis, Davis, CA, USA; ^5^ University of California San Francisco School of Medicine, San Francisco, CA, USA; ^6^ Kaiser Permanente Northern California Division of Research, Pleasanton, CA, USA

## Abstract

**Background:**

Studies on sex/gender differences in dementia risk have often overlooked racial and ethnic differences, particularly within Asian American communities. Our aim is to compare sex/gender‐specific lifetime dementia risk conditional on dementia‐free survival to age 60 among Chinese, Filipino, Japanese, South Asian, and non‐Latino White older adults in California.

**Method:**

We leveraged a cohort study including mailed surveys linked with electronic health records (EHR) (2002‐2020) from Kaiser Permanente Northern California (KPNC). These analyses included survey respondents ages ≥60 years who identified as Chinese, Filipino, Japanese, South Asian, or non‐Latino White with at least two years of KPNC coverage and no dementia diagnosis at time of survey. Sex/gender and dementia diagnoses were ascertained from the EHR. We used the Aalen‐Johansen estimator to calculate the cause‐specific cumulative incidence of dementia conditional on dementia‐free survival to age 60 (i.e., lifetime dementia risk), considering death as a competing event, and calculated sex/gender dementia risk differences and risk ratios at ages 75, 80, 85, 90, and 95.

**Result:**

We included 159,477 participants who self‐identified as follows: 6,415 as Chinese, 5,020 as Filipino, 3,314 as Japanese, 1,061 as South Asian, and 143,667 as non‐Latino White. The proportion of women ranged from 39% (South Asian) to 63% (Japanese); mean baseline age ranged from 68 (South Asian) to 73 years (Japanese). Among women, lifetime dementia risk ranged from 38% (95%CI: 38‐39) (Non‐Latino White participants) to 49% (95%CI: 40‐67) (South Asian participants); among men, lifetime dementia risk ranged from 28% (95%CI: 25‐31 Chinese participants; 95%CI: 22‐37 South Asian participants) to 35% (95%CI: 30‐39) (Japanese participants) (Figure 1). The largest sex/gender differences in lifetime dementia risk were observed among South Asian and Chinese participants (Figure 2). Absolute sex/gender differences in dementia risk emerged at age 80 for South Asian participants, age 85 for Chinese, Japanese, and non‐Latino White participants, and age 95 for Filipino participants. Overall, dementia‐free mortality was substantially higher for men than women.

**Conclusion:**

Lifetime dementia risk conditional on dementia‐free survival to age 60 was higher among women than men and varied across racial and ethnic groups, potentially driven by differences in mortality and social and structural factors.